# Refining Our Focus by Bridging Theory and Practice for Real-World Impact: An Updated Scope for JMIR Medical Informatics

**DOI:** 10.2196/109008

**Published:** 2026-09-04

**Authors:** Arriel Benis, Amanda Iannaccio, Andrew J Coristine, Tiffany I Leung

**Affiliations:** 1 Department of Biomedical Engineering, Duke University, Durham, NC, United States; 2 Department of Biomedical Informatics, Jacobs School of Medicine and Biomedical Sciences, University at Buffalo, Buffalo, NY, United States; 3 JMIR Publications, Inc, 130 Queens Quay East, Unit 1100, Toronto, ON, M5A 0P6, Canada, 1 416-583-2040; 4 Division of Cardiology (affiliate), Department of Medicine, McGill University, Montreal, QC, Canada; 5 Department of Internal Medicine (adjunct), Southern Illinois University School of Medicine, Springfield, IL, United States

**Keywords:** medical informatics, biomedical informatics, informatics applications, informatics, clinical applications, artificial intelligence, digital health applications

## Abstract

To align with contemporary trends and place greater focus on persistent and novel challenges in the field, *JMIR Medical Informatics* has updated its focus and scope. Each submitted work will be evaluated against a stronger criterion for translational impact. Additionally, submissions on AI methods and applications are expected to meet standards of rigor and reporting transparency. In this editorial, we explain further what changed; why; and what this means for prospective authors, reviewers, and readers.

## Introduction

The pace of technological advancements and applications in medical informatics continues to accelerate. Scientific, peer-reviewed publishing is also evolving in parallel. To align with contemporary trends and bring greater focus to persistent and novel challenges in the field, *JMIR Medical Informatics* has updated its editorial scope and focus. In this editorial, we explain what changed; why; and what this means for prospective authors, reviewers, and readers. As biomedical and health informatics (BMHI) has evolved, so has the process and production of research and publications. To maintain priority and focus on high-quality research with translational impact and rigorously conducted and reported AI applications, we outline the editors’ vision for updating the journal’s focus and scope.

## What Has Changed

The core identity of the journal is unchanged: *JMIR Medical Informatics* remains a home for high-quality research on BMHI and, specifically, medical informatics; clinical informatics applications; and the full pipeline of data on medicine, health, and well-being—from acquisition and semantics through analytics, decision support, and evaluation. The journal’s updated scope provides guidance for two specific groups of submissions to *JMIR Medical Informatics*.

*First, each submitted work will be evaluated against a stronger criterion for translational impact. JMIR Medical Informatics* focuses on applied, translational research and implementation science. The revised scope explicitly prioritizes “research that bridges theoretical frameworks with actionable insights, ensuring that informatics solutions demonstrate measurable clinical or population impact” [1]. Theoretical frameworks should be accompanied by actionable insights. To meet this updated expectation, authors must explicitly articulate the clinical and population health implications of their work and should not rely on technical novelty only. Manuscripts should clearly demonstrate how the proposed informatics solutions translate theoretical frameworks into tangible benefits for health systems, care delivery, and patient outcomes.

*Second, submissions on AI methods and applications must state their standards explicitly*. Submitted work is expected to provide evidence of clinical applicability and maturity. This includes all submitted manuscripts that build on machine learning, deep learning, information retrieval, natural language processing, or image processing. The updated journal scope says AI-oriented submissions “must demonstrate utility beyond algorithmic performance on static datasets. Studies should exhibit clinical applicability and maturity, such as integration into care workflows, deployment in routine practice, or robust external validation, with meaningful documented outcomes for patients or clinicians” [1]. Submissions must demonstrate utility beyond algorithmic development or performance on static datasets, including publicly available datasets. While a well-tuned model evaluated only on a held-out test set is valuable, it is no longer sufficient on its own for consideration in this journal. We recommend that authors adhere to the appropriate TRIPOD (Transparent Reporting of a Multivariable Prediction Model for Individual Prognosis or Diagnosis) guidelines, such as the TRIPOD+AI or TRIPOD-LLM (large language model) guidelines, for their study design and methodology, with particular attention to item 27c in TRIPOD+AI [2] or item 19g in TRIPOD-LLM [3]; these items underscore the “applicability and generalizability” of the predictive model or LLM [4]. Furthermore, authors should explicitly detail the practice insights and outcomes gained from their deployment, illustrating how the presented tools affect clinical and administrative workflows, reduce cognitive load or burden for health care consumers and providers, and improve operational or health system efficiencies.

*Finally, a complete* Data Availability *statement is expected for all submissions* [5]. Additionally, where the methods involve writing code, authors should include a statement on code availability (eg, making the code available on GitHub).

We also welcome implementation reports [6]—a novel format that was introduced in 2023 as the first of its kind among medical informatics journals and was specifically intended for manuscripts that systematically describe how an informatics solution was translated into a routine clinical environment. Since their inception, implementation reports have followed a complete and standardized reporting guidance [7,8]. Last year, an accompanying i-CHECK-DH–related toolkit was published to further support researchers in carrying out and reporting their work on clinical informatics and digital health implementations [9]. These have become a durable and growing part of our portfolio, and they are exactly the kind of scholarship the revised scope is designed to encourage.

## Why Update Journal Scope

The journal’s scope has been updated to adapt to gradual shifts in submission and publication trends in *JMIR Medical Informatics*. Over the last 2 years, machine learning and deep learning approaches have made up the largest proportion of submissions. Among published articles, those that describe electronic health record infrastructure, interoperability work, and system-level data integration are markedly more common. Additionally, published articles are frequently framed around the implementation and real-world deployment of digital health and informatics. Conversely, submissions that are narrowly focused bibliometric analyses or simple literature reviews (those not applying a rigorous and standardized methodology), as well as submissions on stand-alone generative AI applications, tend not to advance to review or publication.

This pattern aligns with the revised scope: technical sophistication alone, whether in a predictive model or an LLM (or any other model) application, is not what will earn a place in this journal. The journal prioritizes evidence that a solution has been evaluated in real-world settings by patients, caregivers, clinicians, other health professionals, or decision-makers (eg, within a workflow, health system, or validated deployment). Additionally, we also value transparently reported negative results; rigorously conducted evaluations of implementations that failed to improve clinical or operational outcomes are vital for advancing implementation science and preventing repeated systemic failures, as long as the methodologies and lessons learned are comprehensively documented to prevent the submission of trivial nonresults.

## What This Means for Authors

If your research demonstrates a working BMHI solution or explains why it did not work, we welcome your submission for consideration in *JMIR Medical Informatics*: this is where it belongs. Moving forward, we strongly encourage authors to dedicate a specific portion of their discussion to clinical and informatics practice insights, ensuring that the broader impact on personalized medicine, individuals, populations, public health, and health systems is transparent and actionable. If you are refining a promising model that has not yet left the training environment, we would prefer to see it again once it has matured. We are grateful to the authors, reviewers, and editors who have shaped this journal’s identity, and we look forward to continuing that work together, along with the BMHI communities, as we move forward with the refined focus and scope of *JMIR Medical Informatics*.

## Abbreviations

BMHI: biomedical and health informatics
LLM: large language model
TRIPOD: Transparent Reporting of a Multivariable Prediction Model for Individual Prognosis or Diagnosis
